# Two Cases of Anti-NMDA Receptor Encephalitis

**DOI:** 10.5811/westjem.2016.6.30510

**Published:** 2016-07-21

**Authors:** Jessa Baker, Chris Jeziorkowski, Cory Siebe, Megan Boysen Osborn

**Affiliations:** * University of California, Irvine School of Medicine, Irvine, California; † Advocate Christ Medical Center, Department of Emergency Medicine, Oak Lawn, Illinois; ‡ Denver Health Medical Center, Department of Emergency Medicine, Denver, Colorado; § University of California, Irvine School of Medicine, Department of Emergency Medicine, Irvine, California

## INTRODUCTION

Anti-N-methyl-D-aspartate receptor (anti-NMDAR) encephalitis is a form of autoimmune encephalitis with prominent neuropsychiatric features. Patients present with acute psychosis, memory impairment, dyskinesias, seizures, and/or speech disorders. The clinical course is often complicated by respiratory failure, requiring intubation. Approximately half of patients are found to have an associated ovarian tumor, which expresses NMDAR. Recognition of anti-NMDAR encephalitis by emergency physicians is essential in order to initiate early treatment and avoid psychiatric misdiagnosis. The disease is highly treatable with tumor removal and immunosuppression, and most patients demonstrate a full recovery. In this case series, we report two cases of anti-NMDAR encephalitis in adult women in the United States and provide a review of the literature.

## CASE REPORTS

### Case #1

Chief complaint: “Acting drunk”

A 27-year-old Japanese-American female presented to the emergency department (ED) in August for altered mental status. She had been seen in the ED one and a half weeks prior for fever, diarrhea and vomiting and was discharged home on promethazine. At the time of her second ED visit, her husband described four days of increasing altered mental status and stated that the patient was “acting drunk.” She also had short-term memory deficits, insomnia, hallucinations, falls, and episodes of stiffening and jerking movements. Her past medical, surgical, social, and family histories were negative. She had no recent trauma or zoonotic exposures.

On exam, her vital signs were significant only for a temperature of 38 degrees Celsius rectally and heart rate of 110. She appeared agitated and confused. She had no cervical adenopathy or meningismus. Her pupil exam was normal. Her cardiopulmonary, abdominal, and skin examinations were unremarkable. Despite her confusion, she was alert and moving all extremities. She was uncooperative with the majority of the neurologic exam. Her reflexes, including Babinski’s, were normal. Her speech was clear, but she appeared to be having visual and auditory hallucinations.

Her basic metabolic panel, blood alcohol, urine toxicology screen, thyroid stimulating hormone (TSH), human immunodeficiency virus antibody, acetaminophen and salicylate levels were all within normal limits. Her complete blood count was significant for a white blood cell (WBC) count of 17 x 10^9^ cells/L. Her cerebrospinal fluid (CSF) was significant for 33 white blood cells/μL with 95% lymphocytes, 0 red blood cells/μL, and normal protein and glucose (37 mg/dL, 87 mg/dL, respectively). Her CSF gram stain was negative. A non-contrast head computed tomography (CT) and magnetic resonance imaging (MRI) scan were both normal. A chest x-ray was normal.

The patient was admitted to the hospital for further workup and treatment. She developed intractable seizures and hypoventilation, requiring intubation and sedation. The patient’s CSF was found to be positive for anti-NMDA receptor antibody IgG. Subsequently, a transvaginal ultrasound was performed, which showed a 4 cm ovarian dermoid cyst.

The patient was diagnosed with anti-NMDA receptor encephalitis and improved with immunosuppression and removal of the dermoid cyst.

### Case #2

Chief complaint: “Changes in speech”

A 28-year-old African-American female presented to the ED with changes in speech. She stated that her speech had been “slow” for the past two weeks, associated with an intermittent occipital headache. She denied visual disturbances or focal deficits. Her family members stated that she was having changes in her personality (increased anxiety and tearfulness) and insomnia. Two weeks prior to her current presentation, she had been seen at an outside ED for a “flu-like” illness. Her past medical, surgical, social, and family histories were unremarkable.

On exam, her vital signs were unremarkable. Her head and neck, cardiopulmonary, abdominal, and skin examinations were unremarkable. On neurologic exam, she was alert, oriented, with normal motor and sensation throughout her extremities. Cerebellar exam was within normal limits, including a normal gait. Her speech was slurred with episodes of aphasia. She appeared anxious and was tearful.

Her basic metabolic panel, TSH, and urine toxicology screen were normal. Complete blood count was significant for WBC count of 12.5 × 10^9^ cells/L. Head CT was normal. The patient was admitted and over the next 24 hours, she developed worsening anxiety, mania, paranoia, agitation, auditory and visual hallucinations, left facial droop, and ataxia of the right upper extremity. The admitting team performed a lumbar puncture 36 hours after her initial ED presentation. The patient’s CSF was significant for 320 WBC/μL with 92% lymphocytes, 10 red blood cells/μL, glucose of 50 mg/dL, and protein of 70 mg/dL.

Serum NMDAR antibody IgG was positive. A pelvic ultrasound and pelvic MRI were both negative. The patient was treated with intravenous immunoglobulin (IVIG) and methylprednisolone for three days and had a complete recovery.

## DISCUSSION

Between 1997 and 2007, several authors reported cases of patients with paraneoplastic encephalitis associated with an ovarian teratoma;^1^–^10^ however, the exact etiology of the disease was unknown. In 2007, Dalmau and colleagues discovered anti-N-methyl-D-aspartate receptor (anti-NMDAR) antibodies in the serum and CSF of patients with these distinct neuropsychiatric symptoms.^11^ The disease is now called anti-NMDAR encephalitis and has been widely described. In fact, the California Encephalitis Project found that anti-NMDAR encephalitis was more common than any individual viral etiology of encephalitis in patients younger than 30 years old, exceeding enterovirus, herpes simplex virus, varicella zoster virus, and West Nile virus.^12^

Anti-NMDA receptor encephalitis typically starts with a viral-like prodrome and patients may have headache, fever, nausea, vomiting, diarrhea, anorexia, insomnia, or upper respiratory tract symptoms^11^–^18^ (Table). A few days to weeks later, patients develop psychiatric symptoms (hallucinations, insomnia, fear, catatonia, delusions, mania, paranoia, anxiety, and agitation), short-term memory loss, abnormal movements (orofacial dyskinesias, choreoathetoid movements, muscle rigidity, dystonic postures, oculogyric crisis), seizures, and/or changes in speech (mutism, dysarthria, aphasia, incomprehensible speech).^11^,^12^,^14^–^18^ Other neurologic symptoms such as hemiparesis and ataxia occur less frequently.^11^ On presentation, patients may have a fever.^11^,^13^ As the disease progresses, patients may develop central hypoventilation, requiring intubation, and autonomic instability (hyperthermia, tachycardia, bradycardia, hypertension, hypotension).^13^,^19^

Emergency physicians may be unfamiliar with the clinical presentation of anti-NMDAR encephalitis, since it was not fully characterized in the literature until recently. The diagnosis is often delayed as patients undergo extensive testing (MRI, electroencephalogram, lumbar puncture) and evaluation by neurology and/or psychiatry services to rule out infection or other neurologic or psychiatric cause.^15^,^19^ Many patients get admitted to a psychiatric unit prior to definitive diagnosis.^11^ Head CT is usually normal, but MRI may be abnormal in 25–55% of cases.^20^ Cerebrospinal fluid will be abnormal in 79–100% of cases with elevated WBCs showing a lymphocytic predominance, normal glucose, and normal or elevated protein, with or without oligoclonal bands.^11^,^14^,^16^,^21^ The definitive diagnosis is made through the detection of antibodies to NR1, NR2A and/or NR2B subunits of the NMDA receptor in the CSF or serum.^13^,^14^,^15^,^22^

Approximately one half of patients with anti-NMDAR encephalitis have an associated tumor^14^,^15^,^16^,^19^, most commonly an ovarian teratoma, which, along with the autoimmune nature of the disease, may explain the female predilection for the disorder.^15^,^16^ The frequency of teratomas varies with age, with female patients in the third decade of life having the highest frequency of teratomas.^15^,^16^ These teratomas have been shown to contain both mature and immature neuronal tissue, which expresses NMDA receptor subunits NR1, NR2A and/or NR2B on the surface,^22^ which is thought to be the basis of the antibody response.^11^ Several other tumors have rarely been reported (i.e. testicular teratoma^15^, small-cell lung cancer^15^, neuroblastoma^19^, Hodgkin’s lymphoma^19^), but the majority of patients without an ovarian teratoma have no tumor at all.^14^,^15^,^16^,^19^

Consideration of anti-NMDAR encephalitis by emergency physicians is essential so that early treatment can be initiated. Clinicians should consider the diagnosis in new-onset psychosis or even recurrent psychosis, especially in female patients. The majority of patients will recover with first-line treatment, including tumor removal and immunotherapy (corticosteroids, IVIG, and/or plasmapheresis).^11^,^12^ Dalmau et. al. reported full recovery of eight out of nine patients following tumor resection and immunotherapy.^11^ Refractory cases may respond to rituximab and/or cyclophosphamide.^21^

In conclusion, emergency physicians should suspect anti-NMDAR encephalitis in any young female who presents with new onset seizures, psychiatric symptoms, memory loss, dyskinesias, and/or changes in speech. Patients should be tested for anti-NMDAR subunits in the CSF and serum and should undergo imaging to exclude an ovarian teratoma (ultrasound or MRI). Anti-NMDAR encephalitis is highly treatable with tumor removal and immunosuppression.

## Figures and Tables

**Table:** Incidence of symptoms associated with anti-NMDA receptor encephalitis as described in previous case-studies.

Study and year	Median age and range (years)	Number of patients (Percent female)	Viral prodrome^a^	Psychiatric symptoms^b^	Memory deficits	Changes in speech^c^	Seizures	Movement disorders^d^	Central hypoventilation and/or intubation
Dalmau (2007)^e^	27 (14–44)	12(100% F)	10 (83%)	10 (83%)	6 (50%)	6 (50%)	11 (92%)	7 (58%)	10 (83%)
Iizuka (2008)	26.5(17–33)	4(100% F)	4 (100%)	4 (100%)	NR	4 (100%)	3 (75%)	4 (100%)	3 (75%)
Dalmau (2008)	23(5–76)	100(91% F)	72 (of 84 pts,86%)^f^	77 (77%)	23 (23%)	NR^g^	76(76%)	86 (86%)	66 (66%)
Florance (2009)	14(2–48)	81^h^(85% F)	15 (of 31,48%)	19 (of 32,59%)	NR	17 (of 32,53%)	23 (of 30,77%)	26 (of 31,84%)	7 (of 31,23%)
Gable (2012)	12.5 (2–28)	32(75% F)	NR^j^	≥24^j^ (75%)	NR	23 (72%)	22 (69%)	20 (63%)	13 (41%)
Lin (2014)	18(7–28)	12(83.3% F)	7 (58.3%)	11 (91.6%)	NR	7 (58.3%)	11 (91.6%)	12 (100%)	8 (66.7%)
Wang (2015)	21.6(9–39)	51(63% F)	31(61%)	46 (90%)	16 (31%)	23 (45%)	43 (84%)	29 (57%)	14 (28%)

*F,* female; *GI,* gastrointestinal; *URI*, upper respiratory tract infection; *R,* range; *NR,* not reported

a Viral prodrome includes symptoms such as fever, cough, rhinorrhea, vomiting, diarrhea, headaches.

b Psychiatric symptoms include hallucinations, insomnia, fear, catatonia, delusions, mania, paranoia, anxiety, and agitation.

c Changes in speech include mutism, dysarthria, aphasia, and incomprehensible speech.

d Movement disorders include orofacial dyskinesias, choreoathetoid movements, muscle rigidity, dystonic postures, and oculogyric crisis.

e Dalmau and colleagues include previously reported cases in their case series.

f Information was only available for 84 patients

g Changes in speech described, but exact number was not reported.

h There were 81 patients in the study, but the characteristics of the pediatric patients are those reported.

j The exact number of prodromal symptoms and psychiatric symptoms is not reported. However, 56% of patients had a fever, 28% had GI symptoms, 19% had URI symptoms, and 38% had headache. Hallucinations (66%), psychosis (59%), and irritability (75%) were reported separately, but there was no description of the overall number of patients with psychiatric symptoms.
